# A prospective case series to evaluate subcostal nerve injury with high-resolution ultrasound in posterior retroperitoneoscopic adrenalectomy

**DOI:** 10.1007/s00464-024-10836-5

**Published:** 2024-04-16

**Authors:** Allon van Uitert, Hossein A. Chaman-Baz, Selina E. I. van der Wal, Xiaoye Zhu, Juerd Wijntjes, Henri J. L. M. Timmers, J. Alfred Witjes, Nens van Alfen, Johan F. Langenhuijsen

**Affiliations:** 1 Department of Urology, Radboud University Medical Center, Geert Grooteplein Zuid 10, 6525 GA Nijmegen, The Netherlands; 2 Department of Anesthesiology, Pain and Palliative Care, Radboud University Medical Center, Geert Grooteplein Zuid 10, 6525 GA Nijmegen, The Netherlands; 3 Department of Neurology, Clinical Neuromuscular Imaging Group, Donders Center for Neuroscience, Radboud University Medical Centre, Nijmegen, The Netherlands; 4 Department of Internal Medicine, Radboud University Medical Center, Geert Grooteplein Zuid 10, 6525 GA Nijmegen, The Netherlands

**Keywords:** Adrenalectomy, Retroperitoneoscopic, Subcostal nerve, Nerve damage, High-resolution ultrasound, Case series

## Abstract

**Background:**

Posterior retroperitoneoscopic adrenalectomy has several advantages over transabdominal laparoscopic adrenalectomy regarding operating time, blood loss, postoperative pain, and recovery. However, postoperatively several patients report chronic pain or hypoesthesia. We hypothesized that these symptoms may be the result of damage to the subcostal nerve, because it passes the surgical area.

**Methods:**

A prospective single-center case series was performed in adult patients without preoperative pain or numbness of the abdominal wall who underwent unilateral posterior retroperitoneoscopic adrenalectomy. Patients received pre- and postoperative questionnaires and a high-resolution ultrasound scan of the subcostal nerve and abdominal wall muscles was performed before and directly after surgery. Clinical evaluation at 6 weeks was performed with repeat questionnaires, physical examination, and high-resolution ultrasound. Long-term recovery was evaluated with questionnaires, and photographs from the patients were examined for abdominal wall asymmetry.

**Results:**

A total of 25 patients were included in the study. There were no surgical complications. Preoperative visualization of the subcostal nerve was possible in all patients. At 6 weeks, ultrasound showed nerve damage in 15 patients, with no significant association between nerve damage and postsurgical pain. However, there was a significant association between nerve damage and hypoesthesia (*p* = 0.01), sensory (*p* < 0.001), and motor (*p* < 0.001) dysfunction on physical examination. After a median follow-up of 18 months, 5 patients still experienced either numbness or muscle weakness, and one patient experienced chronic postsurgical pain.

**Conclusion:**

In this exporatory case series the incidence of postoperative damage to the subcostal nerve, both clinically and radiologically, was 60% after posterior retroperitoneoscopic adrenalectomy. There was no association with pain, and the spontaneous recovery rate was high.

**Graphical Abstract:**

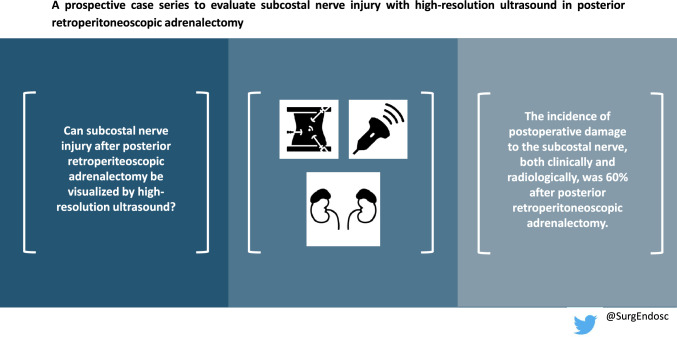

**Supplementary Information:**

The online version contains supplementary material available at 10.1007/s00464-024-10836-5.

In 1994, the posterior retroperitoneoscopic adrenalectomy (PRA) was described as an alternative surgical approach compared to the transperitoneal laparoscopic adrenalectomy (TLA). PRA allows a more direct access to the adrenal gland with minimal dissection of the surrounding structures [1]. Several modifications and refinements of this surgical technique have been described that improve patients’ recovery and reduce the number and severity of postoperative complications [2]. In literature PRA shows excellent results regarding operating times, blood loss, postsurgical pain, and recovery time after surgery [3, 4].

Recently, we performed a large retrospective case series to investigate chronic postsurgical pain and hypoesthesia in patients undergoing minimally invasive adrenalectomy [5]. In this case series, 10% of the patients who underwent PRA reported chronic postsurgical pain and 21% reported hypoesthesia or altered sensations in the abdominal and lumbar skin regions. The presence of chronic postsurgical pain was associated with a significantly lower health-related quality of life compared to patients without pain, whereas the presence of hypoesthesia did not. These findings warranted further investigation.

In the literature chronic postsurgical pain has been reported to be a common complication after surgery, with an incidence ranging from 10 to 50%, depending on the type of surgery [6]. Perioperative nerve injury seems to play an important role in the development of chronic neuropathic pain, since major nerves run through the surgical field in most surgical procedures that are associated with a higher incidence of chronic postsurgical pain [7].

We hypothesized that chronic postsurgical pain and hypoesthesia after PRA may be the result of damage to the subcostal nerve, because it passes the surgical area. The subcostal nerve is a mixed nerve, with sensory branches that supply the skin overlying the lower abdomen (suprapubic region), inguinal region and anterior gluteal region, and motor branches that supply the abdominal muscles (external oblique muscle, internal oblique muscle, transversus abdominis muscle, quadratus lumborum muscle, and pyramidalis muscle) [8]. Therefore, we investigated whether the subcostal nerve could be visualized pre- and postoperatively with high-resolution ultrasound, whether we could visualize nerve injury directly postoperatively, and if there was an association between radiological nerve damage and clinical symptoms and signs after 6 weeks.

## Materials and methods

A prospective single-center case series at the Radboud University Medical Center in Nijmegen was performed between April 2021 and November 2022. An a priori study protocol was written (See Appendix 1) and subsequently approved by the Medical Ethics Committee East-Netherlands (METC Oost-Nederland) CMO-number: 2020-6247. The study was registered in the PaNaMa Research Management System (Mibris, Amsterdam, The Netherlands) with project ID-number: 111037.

### Patient selection

All adult patients who were planned for unilateral PRA were subsequently approached during their urology outpatient clinic visit for inclusion. Patients were eligible for PRA with a body mass index (BMI) of < 35 kg/m2, with a tumor diameter ≤ 7 cm, and with low suspicion of malignancy. Otherwise, TLA or open adrenalectomy was performed. Patients were excluded from participation if they had insufficient understanding of the Dutch language to fill out the questionnaires, if they had undergone previous retroperitoneal surgery, or when they had preoperative (chronic) pain or symptomatic numbness. They were also excluded if they were using preoperative analgesics, anticonvulsants, or antidepressants. Patients with Cushing syndrome were excluded because of their inherent risk factors for delayed wound healing due to a lower quality of subcutaneous fat tissue [9]. Before participating in this study, participants were required to give their informed consent by signing an informed consent form, after being informed of all aspects of the study that were relevant for their decision to participate.

### Preoperative measurements

Baseline characteristics were collected: age, sex, body mass index (BMI), American Society of Anesthesiologists (ASA) score, side of adrenalectomy, duration of surgery, blood loss, perioperative medication, duration of admission, and perioperative surgical complications. Preoperatively, patients were asked to fill out three questionnaires: the McGill Pain Questionnaire to evaluate preoperative pain [10], the self-designed Hypoesthesia questionnaire to evaluate preoperative hypoesthesia [5], and the Pain Catastrophizing Scale questionnaire to evaluate pain catastrophizing [11]. Pain catastrophizing is defined as *‘an exaggerated negative orientation toward actual or anticipated pain experiences’*. A score above 30 in the Pain Catastrophizing Scale represents a clinically relevant level of catastrophizing.

### Perioperative measurements

After admission to the hospital for the surgery, a neurological examination was performed of dermatomes Thoracic-1 (Th-1) to Lumbar-5 (L5) to establish a baseline score regarding sensation of the skin, to be able to compare it with postoperative findings. High-resolution nerve ultrasound was performed or supervised by an experienced neuromuscular ultrasonographer (NvA) using a Sonosite X-porte system (Fujifilm Sonosite, Bothell, USA) with a 6–15 MHz linear probe, directly after induction of general anesthesia and patient positioning in the jackknife position. The subcostal nerve was localized using the position of the tip of the 12th rib and its cross-sectional area (CSA) was measured as close to the tip as possible for standardization [12]. The distance of the nerve to the tip of the 12th rib and its depth from the surface of the skin were measured using the calipers on the machine. Moving the probe more anteriorly the thickness and visual echogenicity of the abdominal muscles (external oblique muscle, internal oblique muscle, transversus abdominis muscle) were measured in the posterior axillary line. The surgeons (JL, XZ) were blinded for the results of these preoperative ultrasound measurements. PRA was performed using the standard three-trocar technique (for example of trocar positions see Appendix 2) and the specimen was removed from the 10 mm port incision. If needed the opening in the fascia was slightly enlarged. After wound closure, a small transparent Tegaderm film (3M™, St. Paul, USA) was applied over the closed trocar entry wounds to guarantee sterility, and the ultrasound measurements of the subcostal nerve and abdominal wall muscles were repeated. The subcostal nerve was specifically assessed for the presence of edema, focal swelling with an increased CSA, and/or a disturbed internal fascicular architecture, which are all signs of nerve damage.

### Follow-up

After 6 weeks, patients were asked to fill out two questionnaires: the McGill Pain Questionnaire to evaluate postoperative pain and the Hypoesthesia questionnaire to evaluate postoperative hypoesthesia. All patients underwent a physical examination by the neurologist (NvA) to assess skin sensation and motor functions of the abdominal wall muscles. If hypoesthesia was found, the exact area was defined and recorded with a digital photography in the electronic patient record. Following the clinical assessment patients underwent a repeat ultrasound to assess for nerve damage, the nerve location in relation to the tip of the 12th rib and skin, the smallest distance of the subcostal nerve to the trocar locations, and the thickness and echogenicity of the abdominal muscles at rest. Comparison to perioperative measurements was made afterward.

After completion of the study, an addition to the study protocol was made to assess long-term effects of surgery and spontaneous recovery of nerve damage. For this purpose, a short, specific questionnaire was created to determine the presence of chronic postsurgical pain, hypoesthesia, and/or muscle weakness (See Appendix 3). After obtaining a repeat informed consent, this questionnaire was sent at the same time to all patients 8 months after the last patient underwent surgery. To minimize the additional burden for the patients, all patients were asked to submit three photographs of their abdominal wall taken from an anterior perspective: one at rest, one during deep inspiration, and one during forced expiration with a Valsalva maneuver, instead of coming to the hospital. These photographs were independently scored by two expert neurologists (NvA, JW), and discrepancies were discussed to achieve consensus about the presence of asymmetry and protrusion of the abdominal wall at rest, and if any lateralisation of the umbilicus to the non-affected side was present during contraction, indicating muscle weakness.

### Statistical analysis

Statistical analysis was performed using SPSS 27.0 for Windows (SPSS Inc., Chicago, USA). Normality was evaluated using the Kolmogorov–Smirnov test. In case of normality, continuous outcomes are displayed as means (± standard deviation), and in case of skewed distribution, outcomes are displayed as median (interquartile range [IQR]). Statistical analysis was performed using Chi-square test, T test, and logistic regression analysis. The significance level was set at 0.05.

This study has been reported in line with the PROCESS Guideline (See Appendix 4) [13].

## Results

### Baseline characteristics

A total of 25 patients, with a mean age of 51.8 ± 9.3 years, were included in this study. Fifty-six percent of the patients were male, and the mean BMI was 26.3 (Table 1). One patient had an incidentaloma, all other patients underwent surgery for primary aldosteronism. Mean tumor size was 16.6 ± 9.3 mm. No patient reported preoperative pain or hypoesthesia of the abdomen, groin, or flank. The median duration of surgery was 59 min (IQR 51–73 min), with a median blood loss of 5 mL (IQR 5–5 mL), and there were no conversions to the transabdominal approach or open surgery. There were no general perioperative surgical complications and median duration of hospital admission was 2 (IQR 2–2) days. There were no differences between left-sided and right-sided adrenalectomy regarding duration of surgery, blood loss, or complications.

**Table 1:** Patient characteristics

	All patients (*n* = 25)
Age during surgery (y)	51.8 ± 9.3
Sex (male)	14 (56)
BMI (kg m^−2^)	26.3 ± 3.8
ASA-score, *n* (%)	
ASA 2	21 (84)
ASA 3	4 (16)
Indication of adrenalectomy, *n* (%)	
Primary aldosteronism	24 (96)
Incidentaloma	1 (4)
Tumor size (mm)	16.6 ± 9.3
Side of adrenalectomy (left / right); n (%)	14 (56)/11 (44)
Duration of surgery (min):	59 (51–73)
Blood loss (mL)	5 (5–5)
Perioperative medications, *n* (%)	
Midazolam	3 (12)
Sufentanil	25 (100)
Propofol	25 (100)
Rocuronium	25 (100)
Lidocaine	13 (52)
Piritramide	19 (76)
Metamizole	22 (88)
Esketamine	8 (32)
Morphine	4 (16)
Postoperative pain medication, *n* (%)	
Paracetamol	25 (100)
NSAID	6 (24)
Oxynorm	17 (68)
Oxycontin	1 (4)
Piritramide PCA	19 (76)
Morphine PCA	4 (16)
Perioperative complications, *n* (%)	0 (0)
Duration of admission (days)	2 (2–2)
Pain Catastrophizing Scale (score)	
Total	6 (29)
Rumination	1 (13)
Magnification	0 (5)
Helplessness	2 (12)

Categorical variables are presented as *n* (%); continuous variables are presented as mean ± SD or median (IQR)

*BMI* body mass index, *y* years, *NSAID* non-steroid anti-inflammatory drug, *PCA* patient-controlled analgesia

### Preoperative measurements

**Fig. 1:**
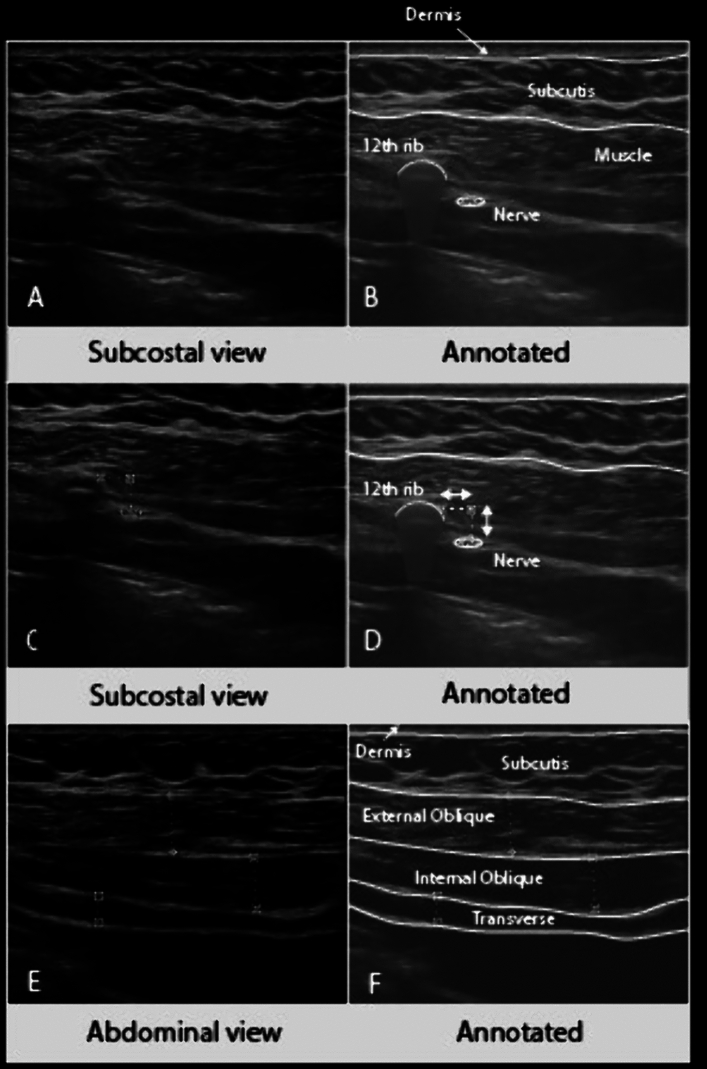
Examples of preoperative high-resolution ultrasound imaging. **A**, **B** Cross-sectional view of the subcostal nerve at the tip of the 12th rib. **C**, **D** Distance of the subcostal nerve to the tip of the 12th rib. (**E**, **F**) Diameters of the abdominal wall muscles at the posterior axillary line

Preoperatively, no patients reported a score > 30 on the Pain Catastrophizing Scale. On preoperative physical examination, one patient had hypersensitivity of the skin in dermatome Th11-Th12 on the ipsilateral side, and one patient had hypoesthesia of the skin in dermatome Th5–Th6 on the ipsilateral side (Table 2). On preoperative CT-scan, three patients had a rudimentary 12th rib; in these cases, the middle trocar was placed at the same level but a few centimeters more laterally to ensure enough space for the medial trocar. Preoperative ultrasound showed a median CSA of 3.0 mm^2^ (IQR 2.0–3.0 mm^2^) of the subcostal nerve at the tip of the 12th rib. The mean distance of the nerve to the tip of the 12th rib was 5.1 ± 2.5 mm craniocaudally and 5.4 ± 2.5 mm in depth (see Fig. 1A–D). The mean diameter of the abdominal muscles was 7.3 ± 2.0 mm for the external oblique muscle, 6.4 ± 2.3 mm for the internal oblique muscle, and 4.7 ± 2.0 mm for the transversus abdominis muscle (see Fig. 1E, F).

**Table 2:** Perioperative study outcomes

Preoperative	
Sensory disturbance on physical exam, *n* (%)	2 (8)
Subcostal nerve, CSA (mm^2^)	3.0 (2.0–3.0)
Distance to tip 12th rib (mm)	
Craniocaudal	5.1 ± 2.5
Depth	5.4 ± 2.5
Abdominal muscles, diameter (mm)	
External oblique muscle	6.9 ± 2.0
Internal oblique muscle	6.7 ± 1.6
Transversus abdominis	4.3 ± 1.9
Directly postoperative	
Subcostal nerve, CSA (mm^2^)	5.0 (3.0—6.0)
Distance to tip 12th rib (mm)	
Craniocaudal	4,7 ± 2.6
Depth	5.9 ± 2.2
Visible nerve damage (edema), *n* (%)	17 (68)
Abdominal muscles, diameter (mm)	
External oblique muscle	7.3 ± 2.0
Internal oblique muscle	6.4 ± 2.3
Transversus abdominis	4.7 ± 2.0

Categorical variables are presented as n (%); continuous variables are presented as mean ± SD or median (IQR)

*CSA cross-sectional area*

### Direct postoperative measurements

The direct postoperative ultrasound measurements were technically difficult to perform due to air artifacts and subcutaneous edema, most likely caused by traction from the trocars and the effects of CO2 insufflation on the local tissue components. In 17 patients (68%), there was evidence of nerve damage with swelling of the subcostal nerve compared to the preoperative scan (median CSA 5.0 mm^2^, (IQR 3.0–6.0 mm^2^). There were no significant differences between the pre- and postoperative diameters of the abdominal muscles.

### Six weeks postoperative measurements

**Fig. 2:**
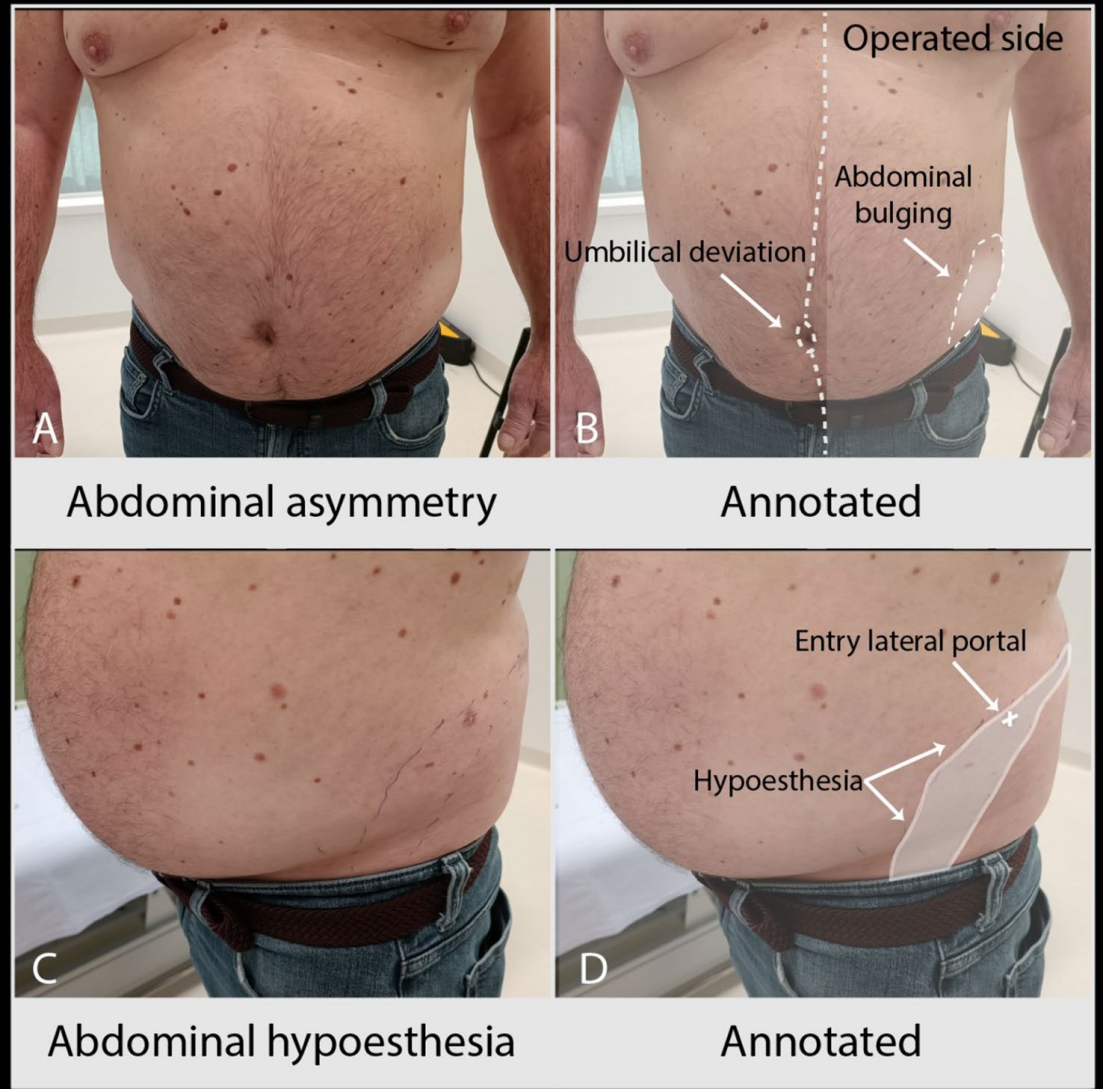
Example of sensory and motor disturbances on physical examination. **A**, **B** Asymmetry of the abdominal wall muscles with ipsilateral muscle bulging and contralateral deviation of the umbilicus. **C**, **D** Area of hypoesthesia of the skin

Six weeks after surgery four patients (16%) reported postsurgical pain, with a mean VAS-score of 3.1. Seven patients (28%) reported numbness or other sensory disturbances, one of whom also reported pain. On physical examination 12 patients (48%) had decreased sensation in dermatome Th10–Th12, of which 11 patients also had weakness of a part of the abdominal muscles (see Fig. 2). Both patients with preoperative hyper- and hypoesthesia of the skin on physical examination had decreased motor functions of the abdominal wall after 6 weeks. Three patients (12%) had muscle weakness without numbness on physical examination.

**Fig. 3:**
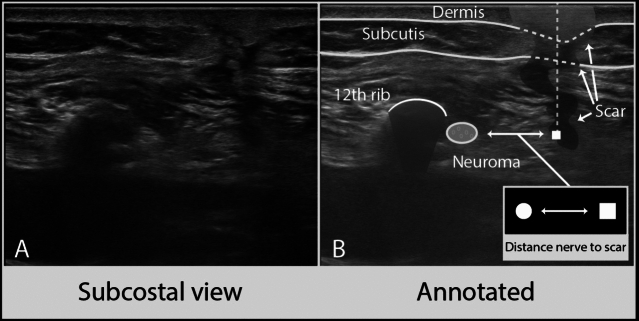
**A**, **B** Example of high-resolution ultrasound after 6 weeks with nerve damage, showing a neuroma of the subcostal nerve and distance to the surgical scar

High-resolution ultrasound of the subcostal nerve showed a neuroma (defined as focal nerve swelling with an increased CSA and a disturbed internal fascicular architecture) in 15 patients (60%) with a mean CSA of 8.6 ± 3.1 mm (see Fig. 3). All patients with numbness or muscle weakness on physical examination had a neuroma on ultrasound (Table 3). All patients with a neuroma reported numbness or muscle weakness, or both.

**Table 3:** Nerve damage after 6 weeks

	Nerve damage (*n* = 15)	No nerve damage (*n* = 10)	*p* value
Age during surgery (y)	55.3 ± 7.7	46.5 ± 9.2	0.02
Sex (male); *n* (%)	8 (53)	6 (60)	0.74
BMI (kg m^−2^)	26.8 ± 3.9	25.6 ± 3.9	0.48
Side of adrenalectomy (left); *n* (%)	7 (47)	7 (70)	0.25
Postsurgical pain, *n* (%)	2 (13)	2 (20)	0.66
Postoperative hypoesthesia, *n* (%)	7 (47)	0 (0)	0.01
Sensory disturbance on physical exam (Th10–12), *n* (%)	12 (80)	0 (0)	< 0.001
Motor disturbance on physical exam, *n* (%)	14 (93)	0 (0)	< 0.001
Both sensory and motor disturbance	11 (73)	0 (0)	< 0.001
Ultrasound measurements after 6 weeks			
Subcostal nerve, CSA (mm^2^)	8.6 ± 3.1	3.3 ± 0.9	< 0.001
Distance to tip 12th rib (mm)			
Craniocaudal	4.8 ± 2.0	3.1 ± 1.7	0.04
Depth	4.9 ± 1.6	4.6 ± 2.1	0.66
Distance subcostal nerve to scar			
Medial trocar, craniocaudal (mm), *n* = 11	22.0 ± 17.8	32.8 ± 10.5	0.23
Medial trocar, depth (mm), *n* = 11	31.0 ± 5.9	24.8 ± 7.8	0.21
Middle trocar, craniocaudal (mm)	11.1 ± 7.1	23.7 ± 22.5	0.05
Middle trocar, depth (mm)	26.4 ± 6.4	24.2 ± 8.8	0.46
Lateral trocar, craniocaudal (mm)	5.9 ± 7.5	12.7 ± 13.2	0.12
Lateral trocar, depth (mm)	25.3 ± 6.9	25.9 ± 11.4	0.86
Abdominal muscles, diameter (mm)			
External oblique muscle	5.4 ± 2.4	7.2 ± 2.6	0.09
Internal oblique muscle	5.4 ± 2.0	6.5 ± 2.8	0.25
Transversus abdominis	3.3 ± 1.0	3.9 ± 1.1	0.18
Abdominal muscles, de novo hyperechogenicity, *n* (%)			
External oblique muscle	4 (27)	1 (10)	0.31
Internal oblique muscle	1 (7)	0 (0)	0.41
Transversus abdominis	3 (20)	0 (0)	0.13
New hyperechogenicity of any muscle	6 (40)	1 (10)	0.10
Long-term measurements, *n* = 21			
Duration after surgery (months)	14.7 ± 6.0	19.0 ± 7.5	0.16
Pain; *n* (%)	0 (0)	1 (11)	0.43
Hypoesthesia; *n* (%)	2 (17)	1 (11)	0.61
Motor disturbance of abdominal muscles; *n* (%)	2 (17)	0 (0)	0.49
Asymmetry on photo (*n* = 18); *n* (%)	6 (55)	4 (57)	0.71

Categorical variables are presented as *n* (%)

Continuous variables are presented as mean ± SD or median (range)

*CSA* cross-sectional area

The mean distance of the subcostal nerve to the most medial trocar scar was 28.9 ± 13.4 mm craniocaudally and 27.1 ± 7.5 mm in depth (see Fig. 3), but this could only be measured in 11 patients as the nerve was not always within the range of the width of the probe between these landmarks. The mean distance of the subcostal nerve to the middle trocar scar was 16.2 ± 16.1 mm craniocaudally and 25.5 ± 7.4 mm in depth, and the mean distance of the subcostal nerve to the lateral trocar scar was 8.7 ± 10.6 mm craniocaudally and 25.6 ± 8.8 mm in depth. The diameters of the abdominal muscles were overall unchanged when compared to the preoperative measurements. There was a de novo hyperechogenicity of the external oblique muscle in 5 patients (20%), of the internal oblique muscle in one patient (4%) and of the transversus abdominis muscle in 3 patients (12%) at 6-week follow-up. There was no difference in the incidence of de novo hyperechogenicity of the abdominal wall muscles between patients with and without nerve damage (*p* = 0.10).

### Long-term effects

Twenty-one patients (84%) completed the long-term follow-up questionnaire, at a median duration of 18 months (IQR 9–24 months) after surgery, four patients were lost to follow-up. One patient reported ipsilateral flank pain, and three patients reported numbness, in two of whom the numb area was close to the scars. All these patients had postoperative nerve damage on physical examination and ultrasound after 6 weeks. In one patient the numbness involved a more extensive area of the ipsilateral flank, hip, and lower back. This patient did not have a neuroma on ultrasound after 6 weeks but had a hematoma with fibrosis in the surgical area. Two patients reported asymmetry of their abdominal wall muscles with bulging of the affected side. Eighteen patients sent photos of their abdominal wall muscles, of which 10 patients had asymmetry (56%). However, the expert assessment of abdominal wall muscle asymmetry using photographs proved to be difficult. When asked specifically, all patients would recommend the surgery to other patients.

### Predictors of nerve damage

There was no significant association between the presence of direct postoperative nerve damage on ultrasound and postsurgical pain, numbness or other sensory symptoms, or muscle weakness at 6 weeks postoperatively. Of the 15 patients with visible nerve damage at 6 weeks, two reported postsurgical pain, and two patients without nerve damage reported postsurgical pain as well (*p* = 0.66). Of the 15 patients with visible nerve damage at 6 weeks, 7 reported numbness (47%), none of the patients without nerve damage reported this symptom (*p* = 0.01). There was a significant association between the presence of nerve damage on ultrasound after 6 weeks and hypoesthesia (*p* < 0.001) or muscle weakness (*p* < 0.001) on physical examination. No patient without nerve damage had sensory or motor symptoms during the physical examination. When looking at the baseline criteria, only high age was significantly associated with nerve damage after 6 weeks (55.4 years versus 46.5 years, *p* = 0.02) (Table 3).

The distance from the subcostal nerve to the tip of the 12th rib was similar in both groups in the preoperative and direct postoperative ultrasound measurements. At 6 weeks, the craniocaudal distance of the subcostal nerve to the tip of the 12th rib was significantly larger in the group with nerve damage (4.8 mm versus 3.1 mm, *p* = 0.04).

## Discussion

In this study the incidence of damage to the subcostal nerve after PRA was high (60%), both clinically and radiologically on high-resolution ultrasound. Historically, three types of nerve damage have been described: neurotmesis (complete transection of the nerve), axonotmesis (disruption of the axons but with an overall intact connective tissue sheath), and neurapraxia (an ischemic or demyelinating impulse block without structural damage) [14]. Common mechanisms of surgery-related nerve injuries include compression, entrapment, direct trauma including transection, crush or laceration injuries, or indirect trauma [15]. The damage to the subcostal nerve in our study could be due to direct damage caused by trocar placement and/or levering of the trocar on the rib needed to position the instruments cranially to reach the adrenal gland, which may cause ischemic, myelin, axonal, and/or connective tissue nerve sheath injury.

Although a nerve injury was visualized in 60% of the patients after 6 weeks and all these patients were symptomatic, most of them recovered spontaneously over the course of 18 months. After long-term follow-up, 1 patient reported pain, 3 patients reported hypoesthesia, and 2 patients had subjective asymmetry of the abdominal wall muscles with bulging. Since the expert assessment of abdominal wall muscle asymmetry using photographs proved to be challenging due to variable photo quality, photo lighting and positioning, and patient obesity, the accuracy of this assessment is not entirely clear.

The subcostal nerve is the largest of the intercostal nerves and provides the most important supply of innervation to the abdominal wall muscles. Compared with the Th11 and L1 intercostal nerves, the subcostal nerve has extensive branching with the other intercostal nerves, which occurs more laterally and anteriorly from the 12th rib in the abdominal wall [16, 17]. This may contribute to the high rate of spontaneous recovery, both sensory and motor, seen in our study even in patients with nerve damage on ultrasound, as reinnervation may occur through the other branches. Furthermore, recovery of the subcostal nerve itself could also play a role when the injury was mild (i.e., neurapraxic or limited axonal loss). This is also seen after spinal surgery and will usually heal within 2–4 months [18]. Because there is an anatomic variability of the 12th rib length and course between patients, this could influence the course and branching pattern of the subcostal nerve as well, since the 12th rib length is associated with variations to the lumbar plexus [19]. In three patients, there was a rudimentary 12th rib and trocar placement was done more lateral from the rib tip. This could influence nerve injury, clinical symptoms, and recovery as well. After open donor nephrectomy with lumbotomy incision, abdominal bulging appeared in 5% of the patients, and recovery rate of abdominal wall muscles measured by ultrasound was seen in all patients after 1 year [20]. Van Ramshorst et al. described a case of abdominal wall paresis after trocar placement for a laparoscopic appendectomy [21], with partial recovery of skin sensation and abdominal wall muscle function after 6 months. Walz et al. showed a prevalence of 8% of chronic hypoesthesia of the abdominal wall after retroperitoneoscopic adrenalectomy for primary adrenal tumors, although the retrospective nature of this study may introduce selection bias into these data [22].

Chronic postsurgical pain is often neuropathic pain and pain with a neuropathic component is usually more severe and persistent than nociceptive pain [23]. The affected nerve and resulting neuropathy, muscle weakness or pain is highly dependent on the type of surgery. After percutaneous nephrolithotomy, sensory neurological complications have been reported in 12% of the patients [24]. After living-donor nephrectomy, the incidence of chronic postsurgical pain was 5.7%, resulting in a significant decrease in quality of life [25]. Although no significant association between postsurgical pain and nerve damage was found in the current study, the incidence of chronic postsurgical pain was lower compared to our previous retrospective data [5]. Therefore, it remains unclear whether subcostal nerve injury is the only culprit for development of chronic postsurgical pain in our patients.

Older age was significantly associated with nerve damage after 6 weeks. It is possible that this could be the result of weakening of the abdominal wall muscles with aging, resulting in more traction on the subcostal nerve, but we cannot substantiate this hypothesis.

In our experience, it was technically challenging to visualize and measure the subcostal nerve directly postoperatively due to air artifacts caused by CO2 insufflation and subcutaneous edema. As the direct postoperative ultrasound findings did not significantly associate with clinical outcomes at 6 weeks, we conclude that this measurement at this time point has limited clinical value.

At 6 weeks, the craniocaudal distance of the subcostal nerve to the tip of the 12th rib was significantly larger in the group with nerve damage (4.8 mm versus 3.1 mm, *p* = 0.04), but did not increase when compared to the preoperative measurements. Furthermore, since there was no difference in craniocaudal distance of the nerve to the 12th rib tip preoperatively and directly postoperatively between the patients with and without a neuroma, it remains questionable whether this is a clinically relevant finding for the surgeon. The mean distance of the nerve to the middle and lateral scar was very small in patients with nerve damage (11.1mm and 5.9mm, respectively), making it a possible cause of the observed nerve damage.

In our study the preoperative course of the subcostal nerve was close to the tip of the 12th rib with a mean distance of 5.1mm craniocaudally. In a cadaveric study by van der Graaf et al., it was shown that while the 10th and 11th intercostal nerves reliably ran flush to the surface of their respective ribs, there was more variability in the course of the subcostal nerve in relation to the 12th rib; it either ran flush with the rib up to a maximum of 3 cm caudally [16]. Currently, the first trocar is placed using the tip of the 12th rib as a landmark and the trocar is placed just caudal to it. However, this could predispose to nerve damage due to the high variability of the course of the subcostal nerve. In our study, the surgeon was blinded for the preoperative ultrasound findings to reduce bias. However, preoperative ultrasound guidance of the surgeon to reduce neurovascular complications has been studied in other fields of surgery [26], so it should be further investigated whether this could be of additional value in PRA as well to avoid direct injury to the subcostal nerve.

There were several limitations to this study. First, there was a short learning curve for the clinical neurophysiologist (NvA) during our study to detect injury to the subcostal nerve, which may have resulted in a higher incidence of postoperative neuromas detected in patients who were included later in the study. Second, long-term nerve recovery was assessed by a questionnaire and photographs, not by repeated ultrasound and clinical examination. Although the patients’ subjective symptoms are the most important outcome factor, the evaluation of the photographs proved to be difficult and therefore, we cannot recommend this method for clinical follow-up. Third, the small sample size could influence the results. Finally, there were 4 patients who were lost to follow-up and could not be included in the long-term results section.

In conclusion, the incidence of postoperative nerve damage after PRA was 60% after 6 weeks. Fortunately, there was no association with postsurgical pain, although there was a significant association with postoperative hypoesthesia and sensory and motor disturbances on physical examination. As all patients with hypoesthesia or muscle weakness on physical examination had a neuroma on ultrasound, physical examination could be used as a primary diagnostic tool when nerve damage is suspected. After 18-month follow-up, the spontaneous recovery rate was high, and only one patient experienced chronic postsurgical pain. Despite our findings, all patients would recommend this surgery to others. Possibly the rate of injury of the subcostal nerve could be reduced by preoperative assessment of the course of the subcostal nerve and subsequent adjustment of the trocar positions to avoid direct damage. However, this needs to be investigated in a prospective manner.

### Supplementary Information

Below is the link to the electronic supplementary material.

Supplementary file1 (DOCX 27 KB)

Supplementary file2 (DOCX 167 KB)

Supplementary file3 (DOCX 269 KB)

Supplementary file4 (DOCX 33 KB)
