# Supplementary material for: A prospective case series to evaluate subcostal nerve injury with high-resolution ultrasound in posterior retroperitoneoscopic adrenalectomy
Source: Surg Endosc. 2024 Apr 16;38(6):3145–55. doi: 10.1007/s00464-024-10836-5 (PMC11133209; doi:10.1007/s00464-024-10836-5)
Supplement: Supplementary file 1 — Supplementary file1 (DOCX 27 KB) [file 464_2024_10836_MOESM1_ESM.docx]

**Research protocol:**

**Effect of retroperitoneoscopic adrenalectomy on the subcostal nerves**

**TABLE OF CONTENTS**

1. INTRODUCTION AND RATIONALE……………………………………………………………………………………………4

2. OBJECTIVES 5

3. STUDY DESIGN 5

4. STUDY POPULATION 5

4.1 Population (base) 5

4.2 Inclusion criteria 5

4.3 Exclusion criteria 5

5. METHODS 5

5.1 Main study parameter/endpoint 5

5.2 Secondary study parameters/endpoints 6

5.2.1 Baseline parameters 6

5.3 Study procedures 6

5.4 Withdrawal of individual subjects 8

6. SAFETY REPORTING 8

6.1 Temporary halt for reasons of subject safety 8

6.2 AEs and SAEs 8

6.2.1 Adverse events (AEs) 8

6.2.2 Serious adverse events (SAEs) 8

6.3 Follow-up of adverse events 8

7. STATISTICAL ANALYSIS 8

7.1 Primary and secondary study parameter(s) 8

8. ETHICAL CONSIDERATIONS 9

8.1 Regulation statement 9

8.2 Recruitment and consent 9

8.3 Benefits and risks assessment 9

8.4 Compensation for injury 9

9. ADMINISTRATIVE ASPECTS, MONITORING AND PUBLICATION 9

9.1 Amendments 9

9.2 Annual progress report 9

9.3 Temporary halt and (prematurely) end of study report 10

9.4 Public disclosure and publication policy 10

10. RISK ANALYSIS 10

11. REFERENCES 10

**SUMMARY**

**Rationale:** The laparoscopic adrenalectomy is the standard of care for benign adrenal tumors. Several modifications and refinements of the surgical technique have been described to improve patients recovery and lower the number of complications, such as the retroperitoneoscopic technique^[[1]](#endnote-1)^. However, 10% of our patients that received a retroperitoneoscopic adrenalectomy report (temporary) pain and 21% (temporary) dullness in the lumbar region, which can be a result of damage to the subcostal nerves (non-published data). We aim to see if the subcostal nerve can be visualized pre- and postoperatively by ultrasound, what the distances are from nerve to trocar locations, and if we can visualise nerve injuries. If so, we aim to see in the future if the surgical technique can be improved to prevent this.

**Objective**: to identify the subcostal nerve in retroperitoneoscopic adrenalectomies and to evaluate perioperative nerve damage

**Study design:** single-centre, prospective cohort study

**Study population:** all patients who undergo a retroperitoneoscopic adrenalectomy, except patients with Cushing syndrome

**Main study parameters/endpoints:**

*Primary objectives:*

- To assess postoperative nerve damage on ultrasound.
- To measure the distances of the subcostal nerve to trocar positions.

*Secondary outcomes:*

- To identify postoperative cutaneous hyperesthesia or hypoesthesia:
- To correlate postoperative dullness, neuropathy, pain or muscle weakness to subcostal nerve damage on ultrasound.

**Nature and extent of the burden and risks associated with participation, benefit and group relatedness:** there are no risks. The burden for the patients is small, due to a longer narcosis (estimated 10 minutes) and extra examinations (ultrasound, physical examination, questionnaire) after 6 weeks.

**INTRODUCTION AND RATIONALE**

In 1992 the first transabominal laparoscopic adrenalectomy (TLA) was described by Gagner et al^[[2]](#endnote-2)^. Compared to the open technique, TLA has advantages with lower morbidity and complication rates, decreased blood loss, less postoperative pain, shorter hospital stay, and improved cosmetic effect^[[3]](#endnote-3)^. Therefore, TLA has become the standard of care for the management of benign adrenal tumors, and in selected cases in the treatment of small malignant adrenal tumors. In 1994 the retroperitoneoscopic adrenalectomy (PRA) was described as an alternative route to the adrenal gland, which allows a more direct access with a minimal dissection of the surrounding structures^[[4]](#endnote-4)^. Already in literature this technique shows excellent results regarding operating times, blood loss, postoperative pain and recovery after surgery^[[5]](#endnote-5)^ ^[[6]](#endnote-6)^.

TLA was introduced in our hospital in 1993. Because of an increase in referred patients, PRA was introduced in our hospital in 2011 for its potential benefits in shorter operating times, less pain, quicker recovery and shorter hospital stay. From 2011, patients were eligible for PRA with a body mass index (BMI) of <35 kg/m2, with a tumor diameter <7 cm, and with low suspicion of malignancy. Otherwise, TLA or open adrenalectomy were performed, for these techniques provide more working space and more landmarks during surgery.

While the rate of postoperative complications after PRA is low, still 10% of our patients report (temporary) pain and 21% (temporary) dullness in the lumbar region, which can be a result of damage to the subcostal nerves. In some cases this is temporary, but in other cases this can be permanent, consequently resulting in a lower quality of life. Our hypothesis is that these symptoms can be the result of damage to the inter- or subcostal nerves (lateral and medial cutaneous branches).

In literature neuropathy is a common symptom after surgery. The affected nerve and resulting neuropathy, muscle weakness or pain depends on different types of surgery. Common mechanisms of surgery related nerve injuries include compression, entrapment/angulation, direct trauma including crushing or laceration injuries, and indirect trauma^[[7]](#endnote-7)^. Walz et al. shows a prevalence of chronic hypoesthesia of the abdominal wall of 8% after retroperitoneoscopic adrenalectomy for primary adrenal tumors, although because of the retrospective character of this study there could be a selection bias in these data^[[8]](#endnote-8)^. After percutaneous nephrolithotomy sensory neurological complications were reported in 12% of the patients, with a significant correlation between prolonged duration of surgery and the incidence of sensory complications^[[9]](#endnote-9)^. After living-donor nephrectomies the incidence of chronic postoperative pain was 5.7%, resulting in a significant lower quality of life^[[10]](#endnote-10)^. Depending on the type of surgery, chronic postsurgical pain is often neuropathic pain (on average 30% of cases with a range from 6% to 54% and more)^[[11]](#endnote-11)^. Pain including such a neuropathic component is usually more severe than nociceptive pain and often affects the quality of life more adversely.

Since the introduction of PRA, several modifications and refinements of the surgical technique have been implemented to improve patients’ recovery and lower the number of intra- and postoperative complications. Because nerve damage can result in significant complaints and this subsequently can result in a lower quality of life, we aim to see if the surgical technique can be further improved by locating this nerve and prevent damage. Therefore, we plan to investigate if the intercostal and subcostal nerves can be visualized pre- and postoperatively by ultrasound, what the distance is of the trocar positions to this nerve, and if we can visualise nerve injuries by ultrasound (direct lesions, oedema). Furthermore, after 6 weeks we want to repeat the ultrasound to see if nerve damage can be visualized at a later stage. If so, we aim to see in the future if the surgical technique can be altered to prevent this.

# OBJECTIVES

*Primary Objective:* to investigate if the subcostal nerve can be visualized perioperatively in patients undergoing retroperitoneoscopic adrenalectomy, to measure distance from the trocar incisions and to assess nerve damage directly postoperative.

*Secondary Objective(s):*

To identify postoperative cutaneous hyperesthesia or hypoesthesia and to correlate postoperative dullness, neuropathy, pain or muscle weakness to subcostal nerve damage on ultrasound, after 6 weeks.

# STUDY DESIGN

A explorative single-center prospective cohort study (n=25) at the Radboud University Medical Centre in Nijmegen.

# STUDY POPULATION

## Population (base)

Adult patients who undergo unilateral retroperitoneoscopic adrenalectomy, except patients with Cushing syndrome at the Radboud University Medical Centre. Patients will be approached at the outpatient clinic to obtain informed consent and participation in this study.

## Inclusion criteria

In order to be eligible to participate in this study, a subject must meet all of the following criteria:

- Age ≥18 years
- Obtained informed consent
- Surgery: unilateral retroperitoneoscopic adrenalectomy

## Exclusion criteria

A potential subject who meets any of the following criteria will be excluded from participation in this study:

- Patients with insufficient control of the Dutch language to fill out the questionnaires.
- Previous retroperitoneal surgery
- Preoperative (chronic) pain
- Preoperative medications: pain medication, anticonvulsants (e.g. pregabaline), antidepressants (e.g. amitriptyline)
- Cushing syndrome (less suitable patient population due to more bad quality fatty tissue and slow wound healing)

# METHODS

## Main study parameter/endpoints

- To assess postoperative nerve damage on ultrasound.
  - - Pre- and postoperative ultrasound to identify and measure the intercostal and subcostal nerves (precise location and cross sectional area (CSA) measurement)
- To measure the distances of the subcostal nerve to trocar positions.
  - - Postoperative measurement of distances (in mm) to trocar positions after marking of the nerve

## Secondary study parameters/endpoints

- To identify cutaneous hyperesthesia or hypoesthesia by:
  - - Physical exam (in collaboration with a pain specialist)
    - Pain Catastrophizing Scale questionnaire ^[[12]](#endnote-12)^

A validated questionnaire about pain catastrophizing (a tendency to misinterpret and exaggerate situations that may be threatening) in 3 subscales: magnification, rumination, and helplessness

- - - Mc Gill Pain Questionnaire, Dutch language version^[[13]](#endnote-13)^

A validated questionnaire about chronic pain, which measures the different qualities of the subjective pain experience

- - - Postoperative ultrasound after 6 weeks with repeat measurements of intercostal and subcostal nerves
- To correlate postoperative dullness, neuropathy, pain or muscle weakness to subcostal nerve damage on ultrasound.

### Baseline parameters

- Date of surgery
- Sex
- Age
- Height
- Weight
- BMI
- Comorbidity
- Medications
- Side of adrenalectomy
- Indication of surgery
- Pressure of pneumo-retroperitoneum
- Duration of surgery
- Intraoperative analgesics
- Postoperative analgesics
- Complications related to PRA
- Length of hospital stay after PRA

## Study procedures

All patients will be approached at the outpatient clinic by AvU (researcher) after the decision is made to perform PRA, explaining the study and its procedures. They will be given additional written information including a consent form. If the patient gives informed consent and has no exclusion criteria, he/she will be included in the trial.

Preoperatively:

1. Preoperative questionnaires: (cross-sectional), duration: in total ± 15 minutes.

- Mc Gill Pain questionnaire (Dutch Language version) (5 minutes)
- Hypoesthesia questionnaire (5 minutes)
- Pain Catastrophizing Scale questionnaire (5 minutes)
- Physical examination (5 minutes)

Intraoperatively:

1. After positioning in jack-knife position, an ultrasound is performed by a clinical neurophysiology research technician with measurement of the intercostal / subcostal nerve (see scan-protocol below). Nerve locations are captured with photography. Since the objective of this explorative study is to identify nerve damage after trocar placement and surgery and to see if the current surgical technique needs improving, surgeon is blinded for the results of preoperative ultrasound measurements to reduce bias.

2. PRA will be performed using the normal, clinically and scientifically validated surgical technique.

3. After wound closure a tegaderm foil is applied over the wound. Then repeat measurement of intercostal / subcostal nerve is performed to assess nerve damage / oedema. The distance of subcostal nerves to trocar locations (shortest distance to nerve, in mm) is measured. Again this is captured with photography. Surgeon is blinded for these results as well to prevent bias in future procedures. After this a sterile drape is applied.

Postoperatively after 6 weeks:

1. Postoperative questionnaires: (cross sectional), duration: in total ± 15 minutes.

- Mc Gill Pain questionnaire (Dutch Language version) (5 minutes)
- Hypoesthesia questionnaire (5 minutes)

2. Physical exam by clinical neurophysiology research technician to evaluate dermatomas and subtle neurological injury

3. Repeat ultrasound measurement of intercostal / subcostal nerve to assess nerve damage if neurological injury is present.

Ultrasound protocol:

- Equipment: Sonoside Xporte ultrasound system with linear 5-16Mhz probe, using preset “zenuwecho S”.

- Positioning of patient: jack-knife (see image below)


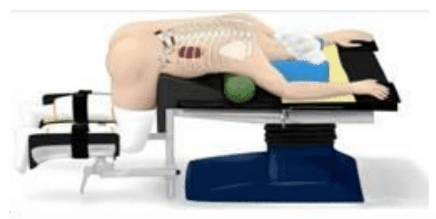


- Scan-protocol:

- Depth: 6cm, gain 60-70%

- Transversal position of probe, midaxillary line. Locating 10^th^, 11^th^ and 12^th^ rib.

- Locating tip 12^th^ rib, locating nerve latero-caudally (power doppler to verify adjacent blood vessel)

- Cross sectional area (CSA) measurement of subcostal nerve, marking the position on skin

- Following lateral / medial cutaneous nerve branches in surgical area, capturing and CSA measurement of abnormalities with note of localization with respect to midaxillary (vertical) and umbilical (horizontal) lines

Data management:

Given informed consent, filled out questionnaires, medical data extracted from electronic patient records are analyzed by AvU and stored in separate folders. Only AvU and HL (primary investigator) have insight in these data. Other authors in this study can view the data upon request. The data will be stored for 15 years.

## Withdrawal of individual subjects

It is a prospective cohort study in which the patients are included after informed consent. Subjects can leave the study at any time for any reason if they wish to do so without any consequences.

# SAFETY REPORTING

## Temporary halt for reasons of subject safety

In accordance to section 10, subsection 4, of the WMO, the sponsor will suspend the study if there is sufficient ground that continuation of the study will jeopardise subject health or safety. The sponsor will notify the accredited METC without undue delay of a temporary halt including the reason for such an action. The study will be suspended pending a further positive decision by the accredited METC. The investigator will take care that all subjects are kept informed.

## AEs and SAEs

### Adverse events (AEs)

Adverse events are defined as any undesirable experience occurring to a subject during the study. All adverse events reported spontaneously by the subject or observed by the investiga­tor or his staff will be recorded.

### Serious adverse events (SAEs)

Not applicable. .

## Follow-up of adverse events

All AEs will be followed until they have abated, or until a stable situation has been reached. Depending on the event, follow up may require additional tests or medical procedures as indicated, and/or referral to the general physician or a medical specialist.

SAEs need to be reported till end of study within the Netherlands, as defined in the protocol.

# STATISTICAL ANALYSIS

The principle investigator (HL) has final responsibility with regard to the data. Incidence of objective and subjective nerve damage will be expressed as absolute numbers and percentages. Distance from trocar to nerve will be expressed in absolute numbers.

## Primary and secondary study parameter(s)

| Study parameters/endpoints | **Presentation of data**  **(quantitative/qualitative)** |
| --- | --- |
| Base-line characteristics | Quantitative |
| Per- and postoperative data | Quantitative |
| Ultrasound parameters | Quantitative |
| Measurement of distance to nerve | Quantitative |
| Postoperative questionnaires | Quantitative |
| Cutaneous hypoesthesia, hypoesthesia, asymmetry abdominal wall after medical examination | Quantitative |

#

# ETHICAL CONSIDERATIONS

## Regulation statement

This study will be conducted according to the principles of the Declaration of Helsinki (version, date, see for the most recent version: www.wma.net) and in accordance with the Medical Research Involving Human Subjects Act (WMO) and other guidelines, regulations and Acts. The data are handled confidentially and anonymously. All subjects will receive a code. Only the study coordinator has access to the source data and the key to the code. When necessary the subject can be identified through contact with the study coordinator. The data will be kept for 15 years after termination of the study. All handling of personal data shall comply with the Dutch Person Data Protection Act (“de Wet Bescherming Persoonsgegevens Wbp”).

## Recruitment and consent

All patients will be approached at the outpatient clinic by AvU (researcher) after the decision is made to perform PRA, explaining the study and its procedures. They will be given additional written information including a consent form. If the patient gives informed consent and has no exclusion criteria, he/she will be included in the trial.

## Benefits and risks assessment

There are no risks. The burden for the patients is small, due to a longer narcosis (estimated 10 minutes) and extra examinations (ultrasound, physical examination, questionnaire) after 6 weeks (estimated 20 minutes).

## Compensation for injury

The sponsor/investigator has a liability insurance, which is in accordance with article 7 of the WMO.

# ADMINISTRATIVE ASPECTS, MONITORING AND PUBLICATION

## Amendments

Amendments are changes made to the research after a favourable opinion by the accredited METC has been given. All amendments will be notified to the METC that gave a favourable opinion.

## Annual progress report

The sponsor/investigator will submit a summary of the progress of the trial to the accredited METC once a year. Information will be provided on the date of inclusion of the first subject, numbers of subjects included and numbers of subjects that have completed the trial, serious adverse events/ serious adverse reactions, other problems, and amendments.

## Temporary halt and (prematurely) end of study report

The investigator/sponsor will notify the accredited METC of the end of the study within a period of 8 weeks. The end of the study is defined as the last patient’s returned questionnaire and/or visited hospital for medical examination.

The sponsor will notify the METC immediately of a temporary halt of the study, including the reason of such an action. In case the study is ended prematurely, the sponsor will notify the accredited METC within 15 days, including the reasons for the premature termination.
Within one year after the end of the study, the investigator/sponsor will submit a final study report with the results of the study, including any publications/abstracts of the study, to the accredited METC.

## Public disclosure and publication policy

The authors have nothing to disclose.

# RISK ANALYSIS

Not applicable.

# REFERENCES

1. Walz et al. Posterior retroperitoneoscopy as a new minimally invasive approach for adrenalectomy: results of 30 adrenalectomies in 27 patients, World J Surg. 1996 Sep;20(7):769-74. [↑](#endnote-ref-1)
2. Gagner M, Lacroix A, Bolte E (1992) Laparoscopic adrenalectomy in Cushing’s syndrome and pheochromocytoma. N Engl J Med 327(14):1033 [↑](#endnote-ref-2)
3. Elfenbein DM et al (2013) Comparison of laparoscopic versus open adrenalectomy: results from American College of Surgeons- National Surgery Quality Improvement Project. J Surg Res 184(1):216–220 [↑](#endnote-ref-3)
4. Gaur DD (1994) Retroperitoneoscopy: the balloon technique. Ann R Coll Surg Engl 76(4):259–263 [↑](#endnote-ref-4)
5. Barczynski M, Konturek A, Nowak W (2014) Randomized clinical trial of posterior retroperitoneoscopic adrenalectomy versus lateral transperitoneal laparoscopic adrenalectomy with a 5-year follow-up. Ann Surg 260(5):740–747 [↑](#endnote-ref-5)
6. Walz MK et al (2006) Posterior retroperitoneoscopic adrenalectomy— results of 560 procedures in 520 patients. Surgery 140(6):943–948 [↑](#endnote-ref-6)
7. Saidha et al (2010) Spectrum of peripheral neuropathies associated with surgical interventions; A neurophysiological assessment, J Brachial Plex Peripher Nerve Inj. 2010; 5: 9 [↑](#endnote-ref-7)
8. Martin K. Walz, M.D., et al., Partial versus Total Adrenalectomy by the Posterior Retroperitoneoscopic Approach: Early and Long-term Results of 325 Consecutive Procedures in Primary Adrenal Neoplasia. World J. Surg., 2004. 28: p. 1323–1329. [↑](#endnote-ref-8)
9. Nasseh et al (2013) Focal neuropathies following percutaneous nephrolithotomy (PCNL) – preliminary study; Ger Med Sci. 2013; 11: [↑](#endnote-ref-9)
10. Bruintjes et al. Chronic pain following laparoscopic living‐donor nephrectomy: Prevalence and impact on quality of life, Am J Transplant. 2019 Oct; 19(10): 2825–2832 [↑](#endnote-ref-10)
11. Arezzo, A., et al., Transperitoneal versus retroperitoneal laparoscopic adrenalectomy for adrenal tumours in adults. Cochrane Database Syst Rev, 2018. 12: p. Cd011668. [↑](#endnote-ref-11)
12. Sullivan et al. (1995). "The Pain Catastrophizing Scale: Development And Validation". Psychological Assessment. 7 (4): 524–532 [↑](#endnote-ref-12)
13. Vanderiet et al. The McGill Pain Questionnaire constructed for the Dutch language (MPQ-DV). Preliminary data concerning reliability and validity. Pain. 1987;30(3):395-408. [↑](#endnote-ref-13)
