# Supplementary material for: A prospective case series to evaluate subcostal nerve injury with high-resolution ultrasound in posterior retroperitoneoscopic adrenalectomy
Source: Surg Endosc. 2024 Apr 16;38(6):3145–55. doi: 10.1007/s00464-024-10836-5 (PMC11133209; doi:10.1007/s00464-024-10836-5)
Supplement: Supplementary file 2 — Supplementary file2 (DOCX 167 KB) [file 464_2024_10836_MOESM2_ESM.docx]

**Appendix 2:**

**Patient positioning with trocar locations**

**
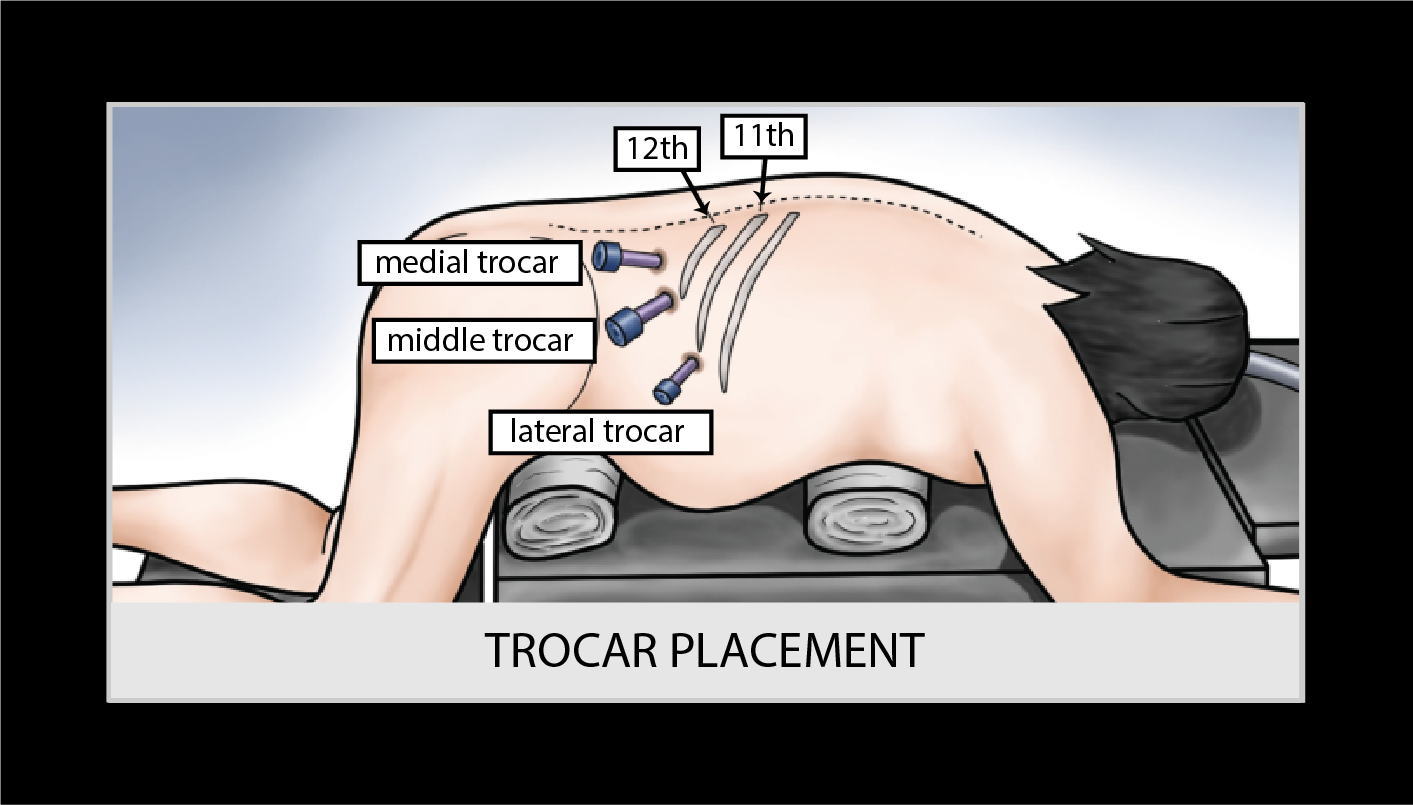
**

**Appendix 2 – legend:**

Example of trocar placement for posterior retroperitoneoscopic adrenalectomy
