# Supplementary material for: A prospective case series to evaluate subcostal nerve injury with high-resolution ultrasound in posterior retroperitoneoscopic adrenalectomy
Source: Surg Endosc. 2024 Apr 16;38(6):3145–55. doi: 10.1007/s00464-024-10836-5 (PMC11133209; doi:10.1007/s00464-024-10836-5)
Supplement: Supplementary file 3 — Supplementary file3 (DOCX 269 KB) [file 464_2024_10836_MOESM3_ESM.docx]

**Appendix 3**

Effect of posterior retroperitoneoscopic adrenalectomy on the subcostal nerve – long term questionnaire

Name:

Date:

Question 1: Do you experience pain at this moment in the surgical area, flank, abdomen or groin? If so, where is this pain located? Can you mark this in the figure and/or describe this in words?


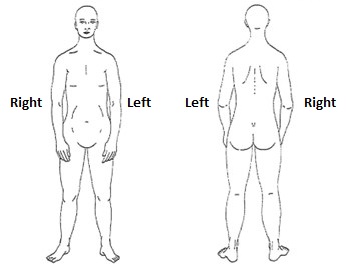


Question 2: Do you experience sensory disorders/hypoesthesia at this moment in the surgical area, flank, abdomen or groin? If so, where is this sensation located? Can you mark this in the figure and/or describe this in words?


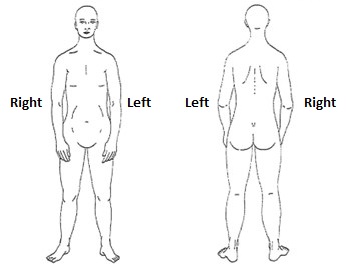


Question 3: Do you experience swelling and/or muscle weakness of the abdominal wall muscles at this moment in the surgical area, flank, abdomen or groin? E.g., during Valsalva manoeuvre, coughing or expiration? If so, where is this located? Can you mark this in the figure and/or describe this in words?


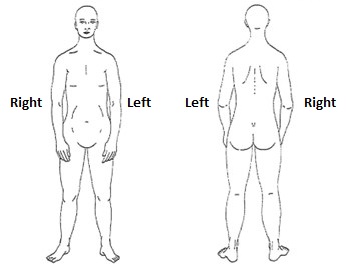


Question 4: Could you send three photos, all made straight from the front, of your upper body and abdomen in the following situations, to allow us to evaluate the function of your abdominal wall muscles?

- Normal, relaxt posture
- After inspiration and holding of breath
- After Valsalva manoeuvre (forced expiration against your hand to engage abdominal wall muscles)

Question 5: Would you recommend this surgery of the adrenal gland to others?
